# Millimeter-wave planar antenna array augmented with a low-cost 3D printed dielectric polarizer for sensing and internet of things (IoT) applications

**DOI:** 10.1038/s41598-023-35707-2

**Published:** 2023-06-14

**Authors:** Yazan Al-Alem, Syed M. Sifat, Yahia M. M. Antar, Ahmed A. Kishk

**Affiliations:** 1 Department of Electrical and Computer Engineering, Royal Military College of Canada, Kingston, ON Canada; 2 Department of Electrical and Computer Engineering, Queen’s University, Kingston, ON Canada; 3 Department of Electrical and Computer Engineering, Concordia University, Montreal, QC Canada

**Keywords:** Electrical and electronic engineering, Electronic and spintronic devices

## Abstract

A unique high gain antenna array with a 3D-printed dielectric polarizer is proposed. The packaging of the antenna array feeding structure is eliminated by aggregating the feeding network in between the antenna elements. This has a significant advantage in maintaining neat and symmetric radiation characteristics with low cross-polarization levels. The proposed structure combines two elements in one feeding point to reduce the array distribution feeding points of a 4 × 4 antenna array from 16 to 8 points. The proposed antenna array structure is extremely low in cost and can be used as either a linearly or circularly polarized one. The antenna array achieves a gain of 20 dBi/dBiC in both scenarios. The matching bandwidth is 4.1%, and the 3-dB Axial Ratio (AR) bandwidth is 6%. The antenna array uses a single substrate layer without the need for any vias. The proposed antenna array suits well various applications at 24 GHz, while maintaining high performance metrics, and low cost. The antenna array can be easily integrated with transceivers due to the use of printed microstrip line technology.

## Introduction

In wireless communications, the channel capacity is proportional to the available bandwidth according to Shannon’s limit. The more bandwidth we have, the higher the capacity of the wireless channel is. As such, a higher data rate can be achieved. By migrating the operation to higher frequencies, such as the anticipated mm-Wave frequencies. The absolute available bandwidth would be significantly larger than the typical RF frequencies. Therefore, higher speeds of wireless communication become attainable^1–3^. While we can have higher speeds at mm-Wave frequencies, the realization of the physical layer becomes more challenging^4–7^. The main disadvantage of operating at higher frequencies is having a higher path loss for the wireless propagating electromagnetic waves once compared with lower RF frequencies. To compensate the path loss, one might suggest increasing the power amplifier gains in the radios. The main issue with this solution is not only that it will drain more power from the supply and cause more heating, but it will also make the devices bulky by accommodating the heat sinks and the required cooling apparatus. For mobile devices, it would be impractical as it will drain the device battery very quickly. A suggested remedy is to use high directivity antennas that will focus the energy towards the communicating entity, this will compensate for the path loss effect and relax the design requirements of the power amplifiers^8–15^.

Operating at mm-Wave frequencies is useful for radar and sensing applications as well. The higher the radar frequency of operations is, the higher the resolution that can be achieved. Several works proposed the use of the 24 GHz mm-Wave band for radar applications^16–20^. The use of short-range radars for health care applications (vital signs sensing)^21^, automotive radar sensors, and motion detectors has become ubiquitous^20,22–26^. Moreover, the developments in wireless connectivity led to the invention of various internet of things technologies. The Internet of Things encompasses plenty of applications. An antenna is an integral part of any IoT communicating device. The performance of these antennas is a crucial factor for the whole system performance. Various antenna structures have been proposed in the Industrial Electronics^27–33^, IoT and Sensors literature^34–41^. In Ref.^42^ a patch antenna with a fence-strip resonator was realized for smart homes IoT communication. For such communication the antenna radiation pattern have to be Omni-directional. In Ref.^43^ a unique glasses frame antenna for IoT communication was realized. A programmable beam scanning antenna without phase shifters was proposed for IoT relay communication in Ref.^24^. In Ref.^26^ a shark-fin antenna was realized, the antenna is to be used for future railway communication systems. In Ref.^23^ a multiband printed smartwatch antenna was proposed, the antenna enhanced the number of frequency bands and improved the Omni-directivity. In Ref.^22^ a microstrip patch antenna was employed in a structural health monitoring (SHM) system to measure structural strain. In Ref.^44^ an electronically steerable parasitic array radiator was used in a dense wireless sensor network. In addition to that, the modelling of antennas is essential in the design process, as an example but not limited to, since the existing antenna equivalent models are inflexible because they assume rectangular antenna contour, a hybrid-equivalent surface-edge current model was proposed in Ref.^45^ to overcome the limitation of the existing models, these models are very useful for vehicle to everything (V2X) communication. Metasurfaces and dispersion engineering techniques can be found to be very useful for various applications as well, Metasurfaces can be utilized to manipulate the characteristics of the propagating waves effectively^46,47^.

The polarization of the antenna is usually determined by the application, for example in satellite communication systems due to the difficulty of alignment, a circular polarization is necessary to avoid any polarization mismatch loss. Radars uses a linear polarization frequently, but they can still use a circular polarization as well^19,48,49^. In this article we present an extremely low-cost antenna array that can be used as either linearly or circularly polarized one. The antenna array has a realized gain of 20 dBi in both scenarios. The matching bandwidth is 4.1% and the AR bandwidth is 6%. The antenna only uses a single substrate layer with no need for any vias. To switch to circular polarization operation, a 3D printed dielectric polarizer is used. The proposed antenna array suits well various applications at 24 GHz, while maintaining high-performance metrics, and extremely low cost. The antenna can be easily integrated with transceivers due to the use of printed microstrip line technology.

## Utilizing microstrip line discontinuities to implement efficient radiators

**Figure 1:**
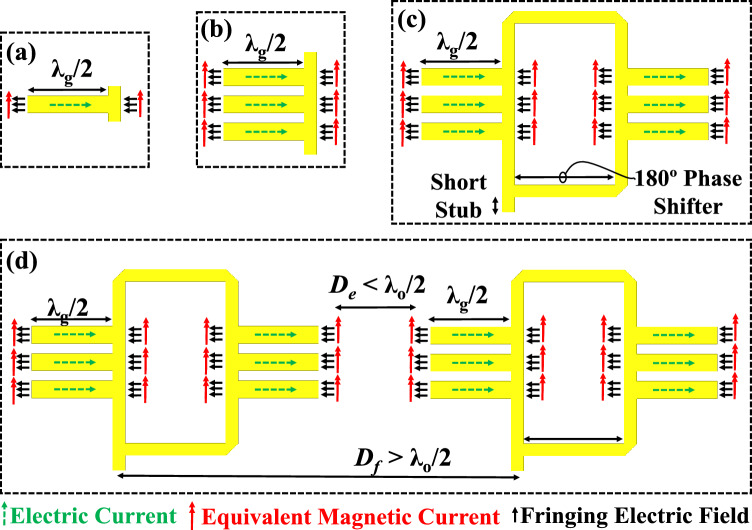
Antenna array element structure, (**a**) Printed microstrip line open stub, (**b**) three printed open stubs in parallel, (**c**) six printed open stubs aggregated in a loop “single antenna array element,” (**d**) two antenna array elements adjacent to each other^55^, λ_g_ is the guided wavelength, and λ_o_ is the free space wavelength.

At higher frequencies, the gain of the antenna is essential to compensate for the path loss; there are several ways and various structures that can be used to achieve high gain performance metric^50^. Reflectors, and lenses are well known for their ability to provide high gain^51^. However they are very large due to their focal length requirement, which makes them harder to integrate. Moreover, they are not considered low-profile solutions. Printed technology is well suited for highly integrated systems, and they are well known for being low profile. Planar arrays can be used to increase the gain performance. By increasing the number of elements in an antenna array, the gain increases proportionally, the rule of the thumb is that by doubling the number of elements the gain increases by 3 dB. However, the issue is that by increasing the number of elements, the associated feeding network size would increase in a proportional manner. The larger the feeding network is, the lager the incorporated loss in it. The total loss in a printed feeding structure is the sum of the dielectric, conduction, and radiation losses. Radiation loss can be reduced by minimizing the radiation produced by the feeding network, ideally a closed structure would suffice to eliminate the radiation loss completely. The dielectric loss can be reduced by using a material with a low loss tangent, ideally vacuum (air). Therefore, closed metallic waveguides are considered to be very efficient feeding structures as they eliminate both radiation and dielectric losses, however they are very expensive and harder to integrate with printed circuits due to their feeding waveguide transition requirement^52,53^. Moreover, by shifting to higher frequencies, the dimensions become smaller and harder for the milling machines to realize. Accordingly, this makes them more prone to tolerance errors, and more challenging to be realized by milling machines. Here, we show that we can maintain a decent efficiency and high gain by using printed technology, while at the same time keeping the structure unpackaged, via-less, and only using a single substrate layer. Figure 1a shows an open microstrip line stub, the open stub is typically used for matching purposes in any printed mircostrip network. Typically, the radiation from these open stubs is minimized as their whole purpose is to alter the input impedance of a certain structure to achieve impedance matching. In Ref.^50^ we showed that such a printed open microstrip line stub can be used as a radiating element where the radiation from the fringing fields can be utilized. As in Fig. 1a, there are two types of currents in this structure, the first is the conduction currents running through the microstrip line, and the second is the equivalent magnetic currents representing the fringing electric fields according to the equivalence principle. The radiation from the conduction currents is suppressed by the image currents running in the opposite direction according to the image theory, this is achieved by ensuring that the ground plane is electrically close to the printed open microstrip line, this feature is desired for the design of printed mircostrip line microwave circuits (i.e., filters, couplers, etc.), such feature minimizes the radiation loss of the intended guiding structure. The magnetic currents radiation is negligible due to the unmatched nature of the structure, where all the input energy is reflected back to the source rather than being radiated. In addition, the aperture area of the fringing fields is very small at the tip of the stub. To utilize the radiation from these open stubs, we need to first increase the aperture area and find a way to match their input impedance to a 50 Ohms source. Figure 1b suggests increasing the aperture area by adding more open stubs, where the aggregated aperture area of all the stubs becomes large enough to achieve significant radiation. Figure 1c suggests having more open stubs on the right side, where the right and left side stubs can be connected by a microstrip line rectangular loop, the loop ensures that the right and left sides magnetic currents are in phase to radiate constructively in the boresight, also it has a significant role in reducing the input impedance to 50 ohms. The element can be perceived as an aggregation of four magnetic current elements, this perception helps in understanding the reason of having suppressed grating lobes for a two-element array despite that the spacing between them is larger than half-wavelength in free space. This can be observed from Fig. 1d, where the sub-element spacing is in the order of a half-wavelength while the two elements spacing is way larger than a half-wavelength. Pattern multiplication can explain this behavior as well, where the high directivity of the element factor knocks down the grating lobes in the array factor. Further detailed analysis for this element can be found in Ref.^54^.

**Figure 2:**
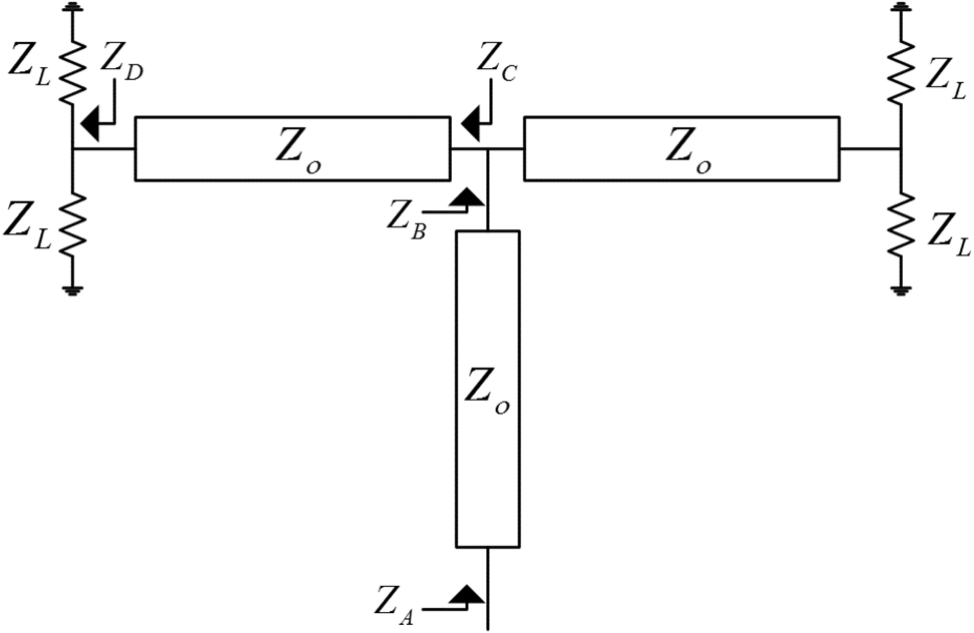
Matching scheme, each transmission line section length is an odd integer multiple of a quarter wavelength.

**Figure 3:**
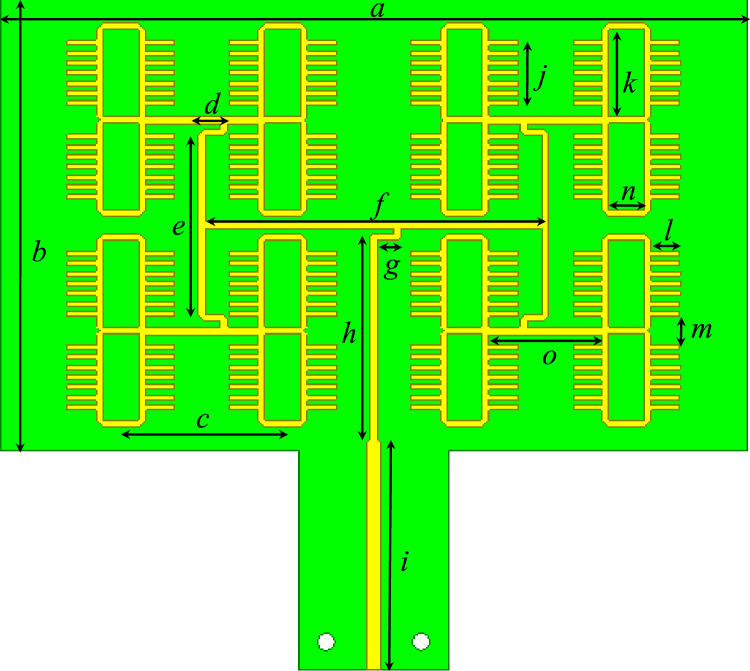
Single-layer printed antenna array utilizing the radiation from open microstrip line stubs (top view).

**Figure 4:**
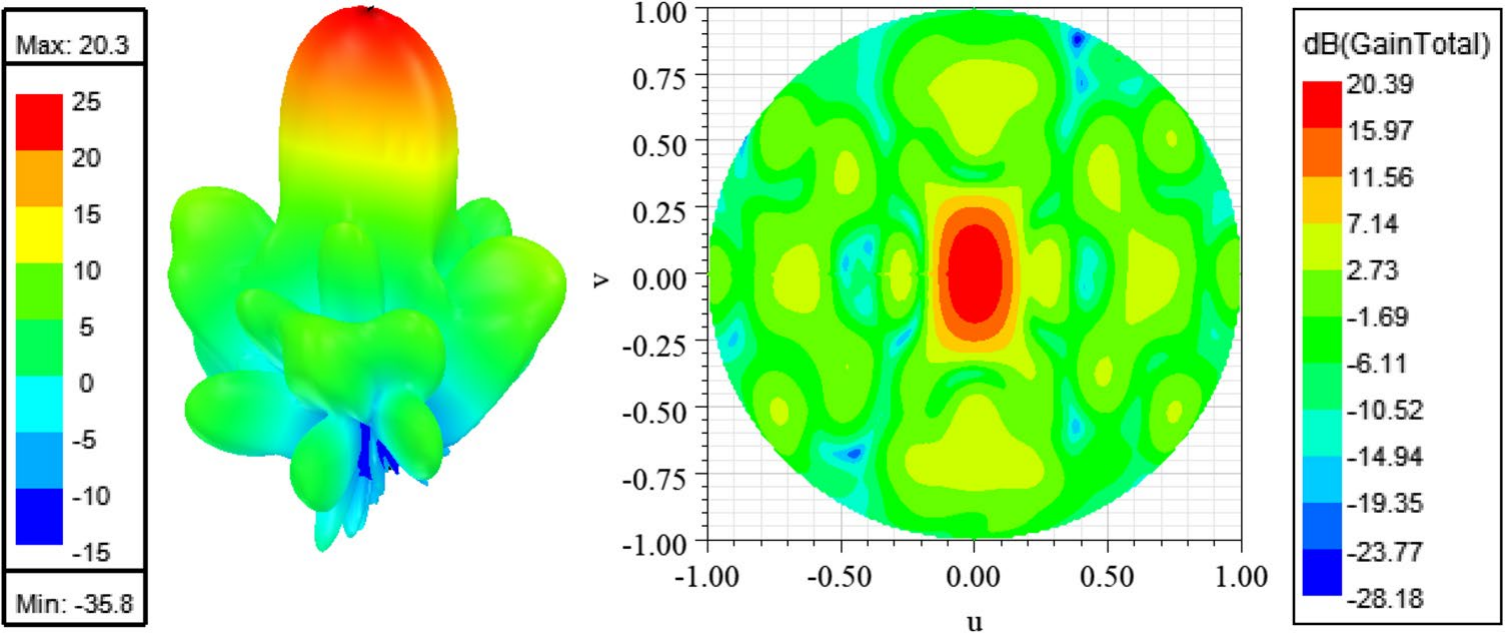
3D radiation pattern “realized gain” (left), and upper sine space (u,v) heat map (right).

The typical procedure to design a feeding network for a 2D array is by using power dividers and quarter wavelength transformers to match the parallel combination of the antenna elements to the desired system impedance. This procedure usually results in transmission lines with different characteristic impedances. Due to the limited space between the antenna array elements, the wide microstrip lines cannot be accommodated, especially in the aggregated configuration. Moreover, very wide microstrip lines can have significant radiation loss^50^. To tackle this issue, the feeding network design procedure in Fig. 2 is proposed. This procedure can provide the required impedance matching by using any arbitrary characteristic impedance. As per the schematic shown in Fig. 2, Eqs. (1–4), and using simple transmission line theory, the input impedance at point “A” will always be equal to Z_L_ as long all the lines lengths are multiple of an odd integer of the guided quarter wavelength. This procedure is very useful where it allows the use of narrow microstrip lines that can fit easily between the radiating elements. Moreover, it minimizes the whole parasitic radiation from the feeding structure due to the use of thin lines which are aggregated between the elements. Further detailed analysis of this concept and comparison cases with packaged structures can be found in Refs.^50,56^. Figure 3 shows a practical realization of this concept. The used substrate is Rogers 5880 with thickness of 0.508 mm, the dielectric constant of the substrate is 2.2, and the loss tangent is 0.0009. Plated vias are not needed at all in the design. The dimensions of the structure are listed in Table 1. At millimeter-wave frequencies, having a packaged guiding feeding structure is highly desirable to eliminate any possible parasitic radiation loss that reduces the antenna efficiency, and affect the radiation characteristics of the antenna. Packaging, or in other words shielding, is usually done by surrounding the feeding structure by a metallic surface. For a planar feeding network, this can be done by shielding the feeding network from the top and bottom by metal sheets. This method is useful in suppressing the radiation from the feeding network. However, this method is undesirable due to the excitation of parallel plate waveguide modes within the shielded package, which eventually constitute a significant source of loss in the feeding structure. Ridge gap waveguide is an example of a new technology that treats such a problem and prevents the propagation of parallel plate waveguide modes^57^. Packaging is a good choice; however, it is not easy to fabricate, and it is more expensive. In the suggested design, the packaging of the antenna array feeding structure is eliminated by aggregating the feeding network in between the antenna elements, this has a significant advantage in maintaining neat and symmetric radiation characteristics with a decent gain in the range of 20 dBi, further details about this technique can be found in Ref.^50^. Figure 4 shows the 3D radiation pattern “realized gain” achieving 20.3 dBi (The calculated results were generated using HFSS^58^).

1 $$Z_{D} = \frac{{Z_{L} }}{2}$$

2 $$Z_{C} = \frac{{Z_{o}^{2} }}{{Z_{D} }}$$

3 $$Z_{B} = \frac{{Z_{o}^{2} }}{{2Z_{D} }}$$

4 $$Z_{A} = 2Z_{D} = Z_{L}$$

**Table 1:** Dimensions (length, width) all in (mm).

***a***	***b***	***c***	***d***	***e***	***f***	***g***	***h***
68.9	45	16.25	3	(17.8, 0.7)	(33.7, 0.7)	3.1	(20.3, 0.7)

***i***	***j***	***k***	***l***	***m***	***n***	***o***	
(22.3, 1.2)	6.5	(8.68, 0.65)	(2.92, 0.5)	2.83	3.65	(11.375, 0.8)

## Dielectric polarizers (additive manufacturing/3D printing)

**Figure 5:**
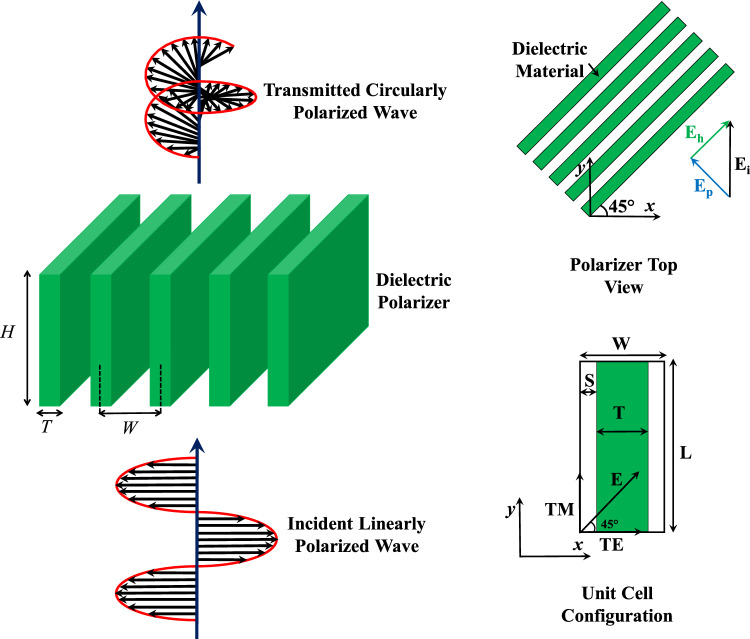
Dielectric polarizer illustration and unit cell analysis configuration.

Simply put, electromagnetic polarizers are structures that are used to transform the wave polarization^59–65^. For example, they can transform a linearly polarized wave to a circularly polarized one and vice versa. Figure 5 illustrates the dielectric polarizer and the unit cell analysis configuration. The polarizer is made of a 3D printed dielectric material with a dielectric constant of 3 and a loss tangent of 0.01. The unit cell analysis is equivalent to an infinite structure of 3D printed dielectric strips. The unit cell analysis assumes a plane wave incidence on an infinite structure, therefore, the designed polarizer dimensions using the unit cell analysis has to be tuned to take into account the waveform of the source (i.e., the antenna), and the fact that the realized dielectric polarizer will be truncated at the edges (i.e., not an infinite structure)^66,67^. The operation of the dielectric polarizer can be explained by decomposing an incident plane wave with 45° angle into two components, parallel and perpendicular or (TE and TM components) as shown in Fig. 5. As such, each component experiences a different effective dielectric constant, and therefore, different phase shift. By controlling the phase shift difference between the two components to be 90°, the linearly polarized wave is transformed into a circularly polarized one. By having a low filling ratio of the dielectric strips, the effective dielectric constants for each component can be approximated as in (5–6)^68^, consequently, the phase difference and the axial ratio can be calculated as in (7–8).

5 $$\varepsilon_{eff\_x} \approx 1$$

6 $$\varepsilon_{eff\_y} \approx 1 + (\varepsilon_{r} - 1)\frac{T}{W}$$

7 $$\Delta \theta = \frac{2\pi f}{c}\left( {\sqrt {\varepsilon_{eff\_y} } - 1} \right)H$$

8 $$AR = \left| {20\log_{10} \left| {\tan \left( {\frac{\Delta \theta }{2}} \right)} \right|} \right|$$

**Figure 6:**
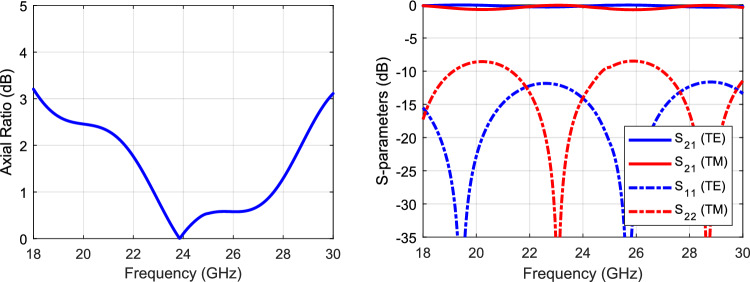
Polarizer axial ratio (left), and S-parameters (right).

**Figure 7:**
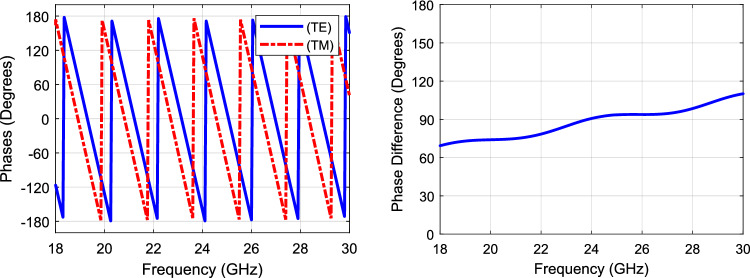
TE and TM phases (left), and phase difference (right).

**Figure 8:**
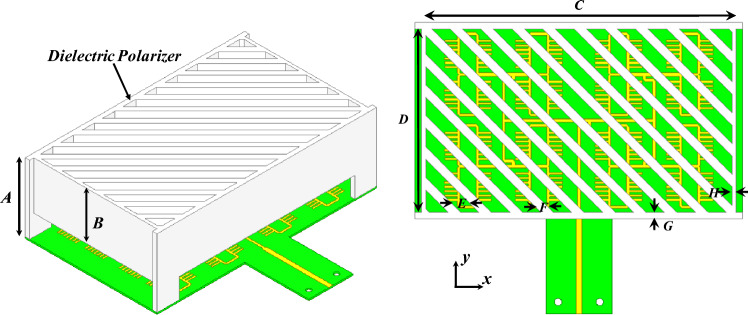
Proposed 3D printed dielectric polarizer antenna array.

**Figure 9:**
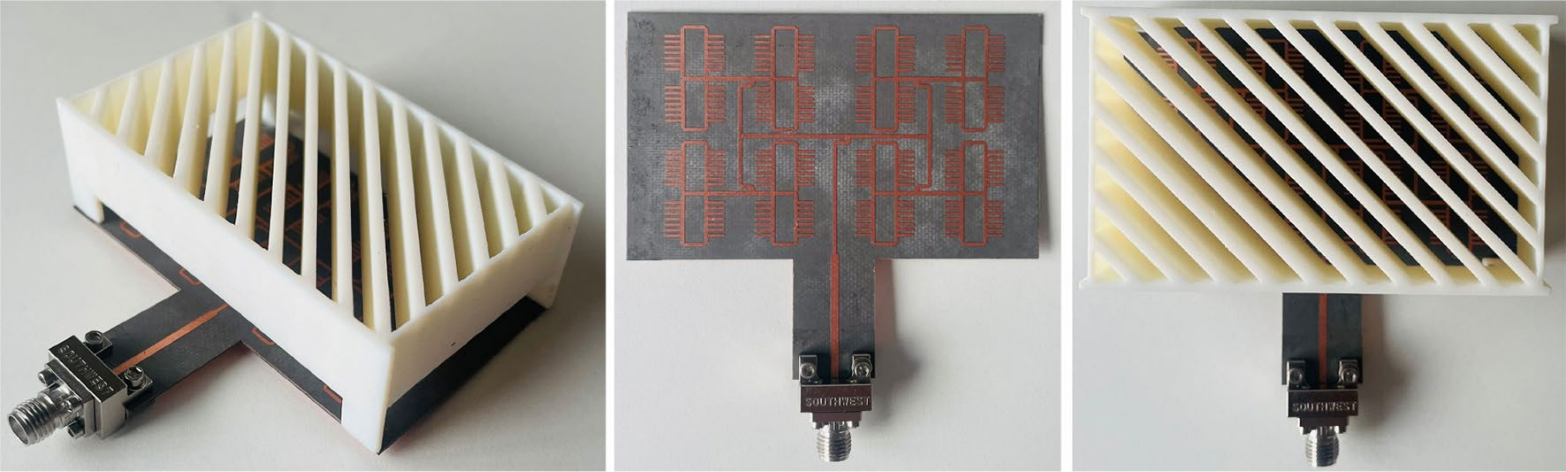
Fabricated prototype.

Table 2 lists the dimensions of the dielectric slab. RGD840 thermoplastics material with dielectric constant value of 3, and loss tangent of 0.01 is used. Figure 6 shows the transmission and reflection coefficients of the two components. As can be seen, there is minimal reflection and almost unity transmission coefficient for each component, indicating almost magnitude equality of the TE and TM electric field components. Hence, the axial ratio can be calculated from the angle difference between these components, as given in (7), the axial ratio is shown in Fig. 6 as well. Figure 7 shows the phase difference between the TE and TM components, and the phase profile for each component. The phase difference is set to be 90° at the center frequency (i.e., 24 GHz) by design. Figure 8 shows the antenna array augmented with the dielectric polarizer; the dimensions of the finite size polarizer are given in Table 3. The array size is 5.15 $\uplambda$_o_ × 3.6 $\uplambda$_o_ and the polarizer height is 1.87 $\uplambda$_o_. Figure 9 shows the fabricated prototype.

**Table 2:** Dimensions, all in (mm), *H* is the dielectric polarizer height.

***W***	***S***	***T***	***H***	***L***
3.32	0.66	2	17.5	6

**Table 3:** Dimensions, all in (mm).

***A***	***B***	***C***	***D***	***E***	***F***	***G***	***H***
23.4	16.4	74.9	42	4.24	2.83	1.5	1.3

**Figure 10:**
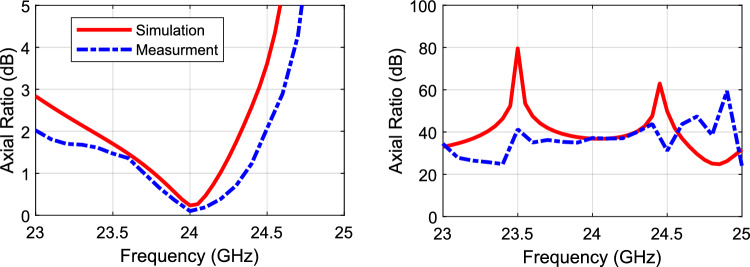
Axial ratio with polarizer (left), and axial ratio without polarizer (right).

**Figure 11:**
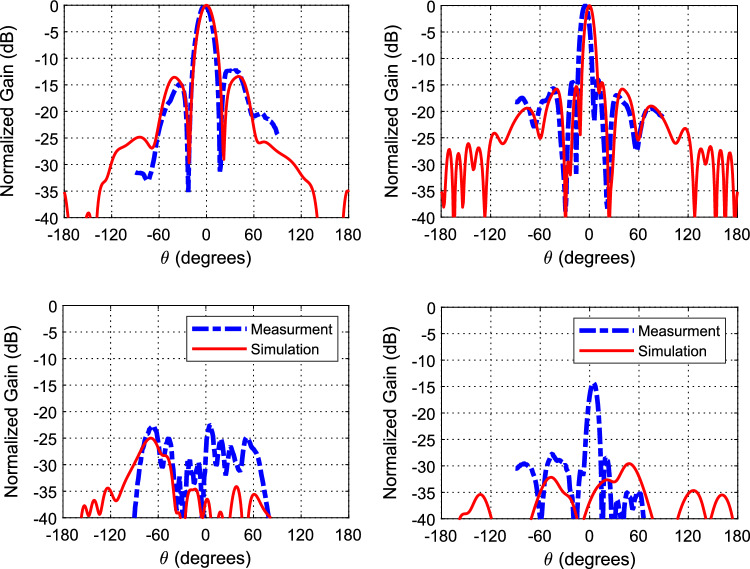
Linear array radiation patterns, H-plane (left), and E-plane (right), co-polar (top), and cross-polar (right).

**Figure 12:**
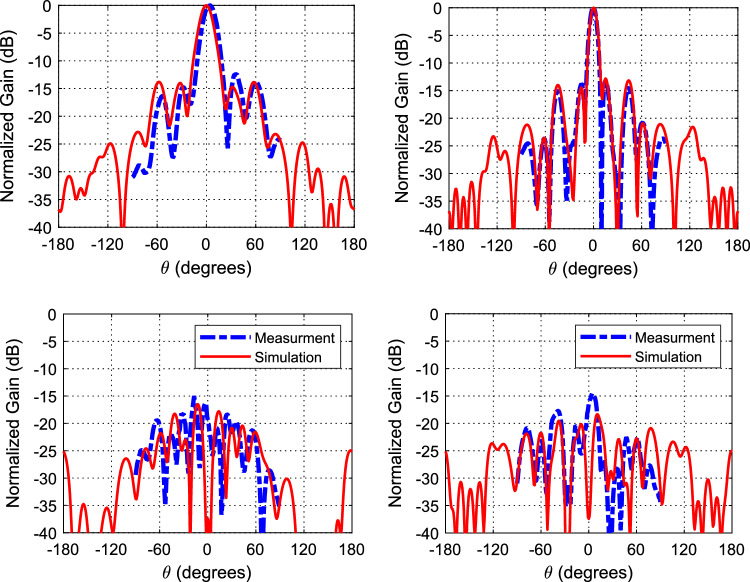
CP radiation patterns, *xz*-plane (left), and *yz*-plane (right), LHCP (top), and RHCP (bottom).

**Figure 13:**
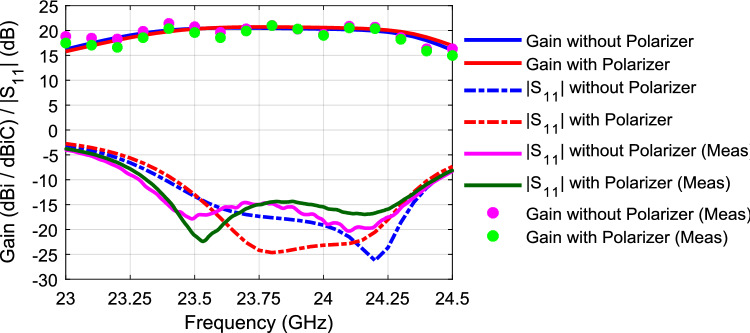
Gain and (reflection coefficient magnitude) |S_11_|.

**Figure 14:**
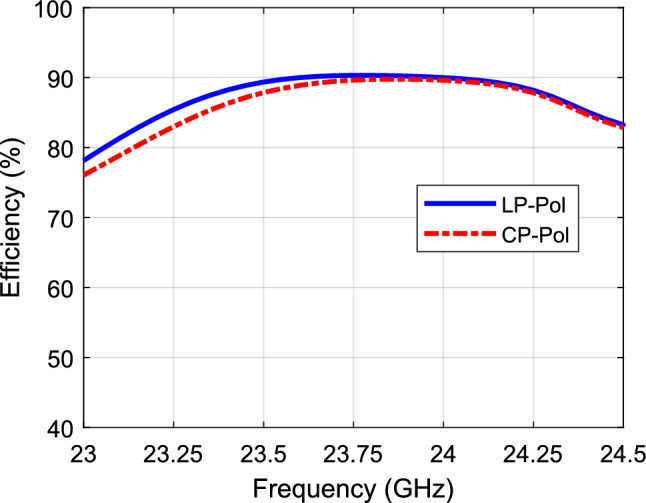
Calculated radiation efficiency.

Figure 10 shows the axial ratio with/without the polarizer. As noted, the axial ratio is below 3-dB over a 6% bandwidth when the polarizer is used. On the other hand, the axial ratio is way above 30 dB when the polarizer is not used (i.e., indicating a linear polarization). Figure 11 shows the radiation patterns in the principal planes, the side lobe level is well below − 14 dB in both planes. Figure 12 shows the radiation patterns in the *xz* and *yz* planes. The radiation pattern shows an LHCP wave in both planes with side lobe level way below − 14 dB. Figure 13 shows the gain value going up to 20 dBi for both cases (i.e., linear, and circular polarization). Figure 14 shows the calculated radiation efficiency. The calculated efficiency is up to 90% in both scenarios as well. The S_11_ in both cases are almost equal indicating a transparent polarizer.

The 20 dBi gain, and the 90% efficiency of the antenna array in both scenarios makes the antenna very attractive for 24 GHz applications (radars, sensors, etc.). The antenna can be used effectively in either mode (CP or LP) depending on the application requirements. While the radiation characteristics encompasses a neat directive pencil beam, the side lobe level is well reduced below − 14 dB in both scenarios. The solution is highly suited for integration with PCB technology. The structure is extremely low in cost where it only uses a single substrate layer with no vias.

**Figure 15:**
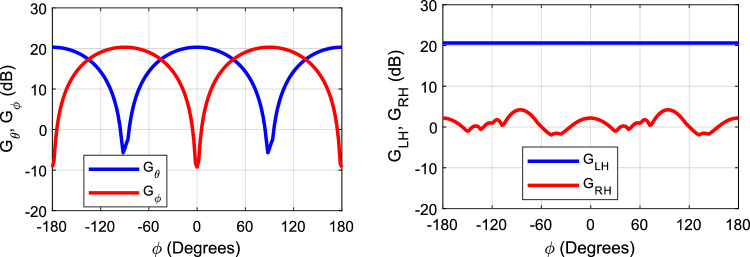
Gain for each polarization component vs azimuthal angle (*ϕ*), linear case (left), and circular case (right).

**Figure 16:**
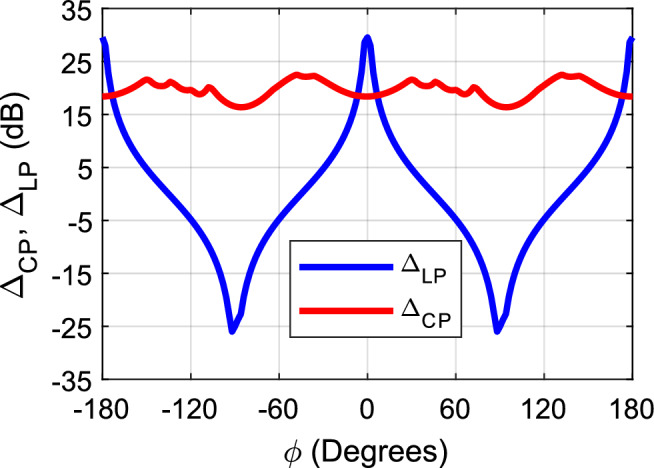
Cross-polar level vs azimuthal angle (*ϕ*) for linear and circular polarization.

It should be clear that there is a slight azimuthal misalignment of the antenna due to the visual alignment of the antenna in the chamber while performing the measurement. The misalignment is noticed from the measured cross-polarization component, which has some contribution from the co-polar in that case. That is why a peak is shown on the broadside of the cross-polarization. Figure 15 shows the gain of each electric field component (E_*θ*_ and E_*ϕ*_) for the linear polarization, and (E_LH_, E_RH_) for the circular polarization. Figure 16 shows the cross-polarization level in each case; the linear case is given by (Δ_LP_ = E_*θ*_ − E_*ϕ*_), and the CP case is given by (Δ_CP_ = E_*LH*_ − E_*RH*_). As expected the circular polarization gain is independent of the azimuthal angle, which makes it immune to misalignment polarization miss match. Also the cross polarization level in the CP case fluctuates from 16.4 to 22.6 dB with the azimuthal angle (i.e. within a 6.2 dB margin). This feature of CP antennas makes it very desirable for several applications which needs an aligned line of sight wireless communication link. On the other hand, the LP antenna shows that the electric field gain depends highly on the azimuthal angle. (N.B. *ϕ* = 0° is the E-plane “*xz*-plane in the CP case”, and *ϕ* = 90° is the H-plane “*yz*-plane in the CP case”). In the E-plane, the co-polar component is E_*θ*_, and the cross-polar component is E_*ϕ*_, and vice versa in the H-plane. Therefore, with the azimuthal misalignment the gain drops from one component and increases in the other component, and the cross polar level can vary from 29.6 to 0 to − 25 dB, the change in the sign represent the switch from one principle plane to the other. Despite the fact that LP antennas are prone to polarization mismatch loss, they well fit several radar applications, this is due to the fact that reflected signals from objects maintain the same polarization (i.e. Linear), and allows the use of one linear antenna for both the transmitter and receiver circuitry which are usually connected through a circulator. On the other hand, in the CP case, the reflected signals from objects flip their polarization (i.e. a LH incident signal will be reflected as a RH signal and vice versa). As such, the transmitting antenna cannot detect the reflected signal, and this will require another antenna with the opposite polarization to detect the reflected signal.

A comparison with other works is shown in Table 4, as the table indicates, the proposed structure is superior in terms of gain (20 dBi/dBiC), and the ability to operate either with circular or linear polarization when compared with other works, unlike other solutions which are optimized to work with one polarization only. The proposed solution maintains a very decent levels for the cross polarization, side lobe level, gain, front to back ratio in both polarizations unlike other works which would compromise some of these performance metrics. In the proposed 4 × 4 antenna array structure, the number of feed points is reduced from 16 to only 8 points by combining two elements with a single feed point. Moreover, the gain of the proposed element is high which makes it easy to achieve 20 dBi/dBiC from a 4 × 4 configuration. This performance is very challenging without using packaging techniques, as an example but not limited to Ref.^69^, an 8 × 8 patch antenna array can only provide 18 dBi of gain.

**Table 4:** Comparison with other works.

References	Freq (GHz)	Array type	Size	BW (%)	Gain (dBi/dBiC)	Polarization	Number of layers	Feed
Ref.^69^	24	Microstrip antenna array	8 × 8	4.1	18	LP only	1	MSL
Ref.^70^	28	SIW based patch antenna array	4 × 4	8.5	19.1	LP only	3	WG
Ref.^61^	30	Patch antenna	1 × 8	11.7	13	LP and CP	2	MSL
Ref.^71^	24.125	Comb array	8 × 8	1.03	19.8	LP only	1	MSL
Ref.^72^	24.125	Patch antenna	1 × 26	2.4	16	LP only	1	MSL
Ref.^73^	28.35	Crossed slot with parasitic patches	4 × 4	14	18.2	CP only	4	WG
Ref.^64^	60	Open waveguide antenna	1 × 1	30	15	CP only	1	WG
Ref.^65^	60	Dielectric lens	1 × 1	29	21.4	CP only	1	WG
Ref.^62^	60	Horn antenna with a lens	1 × 1	32	17	CP only	2	WG
This work	24	Open stub antenna array	4 × 4	4.1	20	LP and CP	1	MSL

The proposed structure only uses a single substrate which makes it extremely low in cost. It doesn’t require any vias, which simplifies the fabrication process significantly. Such simplification in the fabrication is very beneficial, especially at millimeter wave frequencies where the structure/vias dimensions become very small and very susceptible to tolerance errors. Furthermore, the proposed structure uses a single substrate with a MSL feed, which makes it very easy to be integrated with transceiver circuitries. Other works as indicated in Table 4 use a waveguide feed which makes them very bulky. Waveguide feeds also require special transitions to integrate them with typical IC transceivers, these transitions constitute a significant loss that usually has to be compensated with extra gain from the antenna or the power amplifiers.

## Conclusions

A low-cost antenna array operating at 24 GHz was demonstrated, the packaging of the antenna array feeding structure has been eliminated by aggregating the feeding network in between the antenna elements. The proposed antenna array had an extremely low cost and could be used as either a linearly or a circularly polarized antenna array. The antenna array had a realized gain of 20 dBi in both scenarios. The achieved matching bandwidth is 4.1% and the 3-dB axial ratio bandwidth is 6%. The antenna array only used a single substrate layer with no need for any vias. To switch to circular polarization operation, a 3D printed dielectric polarizer was used. The proposed antenna array demonstrated well suitability for various applications at 24 GHz, while maintaining high performance metrics, and extremely low cost. The antenna array could be easily integrated with transceivers due to the use of printed microstrip line technology.

## Data Availability

All data generated or analyzed during this study are included in this published article.
